# Stroke in autoimmune rheumatic diseases

**DOI:** 10.1007/s00296-026-06110-7

**Published:** 2026-04-06

**Authors:** Saltanat K. Yerkebayeva, Meirgul I. Assylbek, Olena Zimba, Yuliya Fedorchenko, Ahmet Usen

**Affiliations:** 1 Department of Neurology, Psychiatry, Rehabilitation and Neurosurgery, South Kazakhstan Medical Academy, Shymkent, Kazakhstan; 2 Department of Social Health Insurance and Public Health, South Kazakhstan Medical Academy, Shymkent, Kazakhstan; 3 Medical Center “Mediker”, Shymkent, Kazakhstan; 4 Department of Rheumatology, Immunology and Internal Medicine, University Hospital in Kraków, Kraków, Poland; 5 National Institute of Geriatrics, Rheumatology and Rehabilitation, Warsaw, Poland; 6 Department of Internal Medicine N2, Danylo Halytsky Lviv National Medical University, Lviv, Ukraine; 7 Department of Pathophysiology, Ivano-Frankivsk National Medical University, Ivano-Frankivsk, Ukraine; 8 Department of Physical Medicine and Rehabilitation, Medipol University Faculty of Medicine, TEM Avrupa Otoyolu Göztepe Çıkışı No: 1, Bagcilar, 34214 Istanbul, Türkiye

**Keywords:** Stroke, Inflammation, Autoimmunity, Rheumatic Diseases, Atherosclerosis, Thrombosis, Risk Factors

## Abstract

Stroke is a major cause of mortality and long-term disability worldwide. Patients with autoimmune rheumatic diseases (ARDs) exhibit a significantly increased risk of both ischemic and hemorrhagic strokes, particularly at younger age. Systemic inflammation, immune-mediated vascular injury, antiphospholipid antibodies, accelerated atherosclerosis, and comorbidities contribute to this elevated cerebrovascular burden. This review aims to comprehensively overview stroke epidemiology, pathophysiological mechanisms, clinical characteristics, prevention strategies, and post-stroke rehabilitation in patients with ARDs. Literature searches were conducted using Medline/PubMed, Scopus, Web of Science, and the Directory of Open Access Journals (DOAJ) databases up to February 1, 2026. Studies evaluating stroke incidence, risk factors, mechanistic pathways, prevention approaches, and rehabilitation in rheumatoid arthritis, systemic lupus erythematosus, systemic sclerosis, ankylosing spondyloarthritis, systemic vasculitides, and Behçet disease were included. Stroke risk is markedly increased across ARDs, with relative risks ranging from 1.3 to 2.5, depending on disease subtype. Systemic lupus erythematosus and systemic vasculitides confer particularly high cerebrovascular risk, often presenting at younger age and associated with worse functional outcomes. Chronic systemic inflammation, endothelial dysfunction, autoantibody-mediated thrombosis, genetic susceptibility, and accelerated atherosclerosis represent central mechanistic pathways. Certain immunomodulatory therapies may mitigate stroke risk through inflammation control, whereas others require careful cardiovascular risk assessment. Post-stroke recovery may be adversely influenced by persistent inflammatory activity and musculoskeletal comorbidities, underscoring the importance of multidisciplinary management. Early risk stratification, tight control of systemic inflammation, individualized immunomodulatory therapy, and structured rehabilitation strategies are essential to improve cerebrovascular outcomes in ARDs. Future research should focus on personalized risk prediction models and targeted preventive interventions in this high-risk population.

## Introduction

Stroke is a leading cause of mortality and long-term disability worldwide [1]. According to the Global Burden of Disease Study (1990–2021), stroke is the third leading cause of death globally, accounting for 10.7% of all deaths [2]. It is also ranked fourth in terms of disability-adjusted life years (DALYs) [2].

Stroke is generally considered a disease of later life, with first-ever events in high-income countries occurring at a median age above 70 years, and only 5–15% manifesting before 55 years [3]. In low- and middle-income countries, stroke onset is reported approximately five years earlier and disproportionately affects younger adults, accounting for 14–24% of cases [3].

Patients with autoimmune rheumatic diseases (ARDs) experience elevated stroke risk relative to the general population, particularly in younger individuals [4]. The highest proportional risk is observed in patients younger than 50 years (Odds Ratio [OR] 1.79, 95% CI 1.46–2.2), while the excess risk is attenuated in those older than 65 years (OR 1.14, 95% CI 0.94–1.38) [4]. Pooled analyses indicate substantial increases in ischemic and hemorrhagic strokes among individuals with rheumatoid arthritis (RA) (OR 1.64–1.68) and systemic lupus erythematosus (SLE) (OR 1.82–2.11) [4].

Rheumatic disease–related stroke risk is multifactorial. In RA, systemic inflammation is a key driver, with elevated erythrocyte sedimentation rate (ESR) associated with ischemic stroke and accelerated atherosclerosis [5]. Established cardiovascular risk factors, along with extra-articular involvement, amplify stroke risk [5]. Suppression of systemic inflammation is therefore crucial to reduce vascular morbidity [6]. RA-specific risk stratification, combined with lifestyle modifications, minimized corticosteroid/NSAID use, and early DMARD therapy, optimizes metabolic and lipid profiles while lowering the incidence of vascular complications, including atrial fibrillation [6].

Stroke prevalence in SLE ranges from 2 to 19%, with incidence rates of 5.8–25.3 per 1,000 person-years [7]. Antiphospholipid antibodies (APA), particularly lupus anticoagulant (LAC), are strongly associated with ischemic stroke [5]. SLE-related strokes tend to occur early after diagnosing SLE due to systemic inflammation, endothelial dysfunction, and APA-mediated hypercoagulability [7].

Epidemiological data reveal increased stroke risk across all types of arthritis [8]. Arthritis is associated with a markedly elevated risk of stroke, both after adjustment for age and sex (RR = 1.36, 95% CI 1.27–1.46) and for at least one established cardiovascular risk factor (RR = 1.4, 95% CI 1.28–1.54) [8].Stroke risk is high in RA (RR = 1.38), ankylosing spondylitis (RR = 1.49), psoriatic arthritis (RR = 1.33), and gout (RR = 1.4) [8].

Young-onset ischemic stroke is markedly elevated in autoimmune inflammatory rheumatic diseases (AIIRDs) [9]. In a Taiwanese cohort, 223 (0.8%) patients with AIIRDs versus 1,923 (0.3%) controls experienced stroke before 50 years [9]. Incidence per 100 person-years was highest in systemic vasculitis (0.44), SLE (0.26), and systemic sclerosis (0.24) versus 0.05 in the general population [9].

Inflammatory mechanisms common to autoimmune and infectious disorders contribute to ischemic stroke by inducing vascular injury [10]. Circulating markers of inflammation, including CRP and IL-6, are consistently linked to stroke occurrence, recurrence, and adverse post-stroke outcomes [10].

The aim of this review is to comprehensively overview stroke characteristics, underlying pathophysiological mechanisms, prevention strategies, and post-stroke rehabilitation in patients with ARDs.

## Search strategy

Comprehensive literature searches were conducted in Medline/PubMed, Scopus, Web of Science, and the Directory of Open Access Journals (DOAJ) to identify studies of stroke incidence, risk factors, pathophysiological mechanisms, prevention strategies, and post-stroke rehabilitation in patients with ARDs. Eligible study designs included systematic reviews, meta-analyses, clinical trials, cohort studies, case–control studies, observational studies, case series, and case reports published up to February 1, 2026. Search terms combined disease-specific and outcome-related keywords using Boolean operators: (“rheumatoid arthritis” OR “systemic lupus erythematosus” OR “systemic sclerosis” OR “ankylosing spondyloarthritis” OR “psoriatic arthritis” OR “vasculitis” OR “Behçet disease” OR “autoimmune rheumatic disease”) AND (“stroke” OR “ischemic stroke” OR “hemorrhagic stroke” OR “cerebrovascular event” OR “transient ischemic attack” OR “cerebrovascular complication”) AND (“risk factor” OR “pathophysiology” OR “inflammation” OR “vascular injury” OR “prevention” OR “rehabilitation” OR “functional recovery” OR “biomarkers” OR “imaging” OR “artificial intelligence”). Only English articles were analyzed. The search strategy, screening, and selection of studies adhered to guidelines for comprehensive literature reviews [11].

### Systemic lupus erythematosus

SLE is an autoimmune disease characterized by multisystem affections and predisposition to vascular complications [12]. Accumulating evidence demonstrates that SLE confers a markedly elevated cerebrovascular risk [12, 13] A meta-analysis of 46 studies reported more than a twofold increased risk of stroke in SLE (RR 2.5; 95% CI 2–3.1), with an absolute stroke risk of 3% and an incidence of 4.72 per 1,000 person-years, particularly affecting younger individuals [13]. In a retrospective cohort of 8,310 SLE patients and 16,620 controls, adjusted analyses showed increased hazards for ischaemic stroke (adjusted Hazards Ratio [aHR] = 1.65, 95% CI 1.45–1.87) and haemorrhagic stroke (aHR = 2.24, 95% CI 1.71–2.95) [14].

Mendelian randomization analyses demonstrated a significant association between SLE and ischemic stroke (OR 1.039, 95% CI 1.014–1.066) [15]. Integrated transcriptomic studies revealed shared immunogenetic signatures and common driver genes—including EIF2AK2, PARP9, and IFI27—suggesting overlapping pathogenic pathways in ischemic stroke and SLE, mediated by immune dysregulation and neuroinflammation [16].

Cerebrovascular events in SLE encompass diverse subtypes. In a retrospective SLE cohort, ischemic stroke was most frequent (58.3%), followed by subarachnoid hemorrhage, cerebral venous thrombosis (CVT), intracerebral hemorrhage, and transient ischemic attack [17]. Ischemic events exhibit a bimodal temporal distribution—occurring early (≤ 1 year) and late (> 10 years) after SLE diagnosis—whereas intracerebral hemorrhage more commonly presents later in the disease course [17]. Antiphospholipid syndrome (APS) and aneurysmal rupture represent major associated factors [17, 18]. Extracranial arterial pathology—including carotid dissection and vasculitis—also contributes to stroke pathogenesis, necessitating vascular imaging with contrast-enhanced Magnetic Resonance Angiography (MRA) and Doppler ultrasound, and catheter angiography in selected cases [7].

Stroke can be the initial manifestation in young women with SLE [19, 20]. Sex-specific patterns are evident in young stroke cohorts, where SLE predominantly affects women [21]. Retinal vasculitis is an independent predictor of stroke in SLE (HR 2.25) [22–24].

Importantly, stroke in SLE carries worse long-term consequences compared with the general population [25]. SLE independently predicts poor 90-day functional outcomes (OR 5.4), higher stroke recurrence (30%), increased post-stroke seizures, and substantially greater long-term mortality (37.5%) [25]. Large nationwide cohort studies demonstrate increased in-hospital mortality and long-term recurrent cerebrovascular events in lupus patients with ischemic and hemorrhagic strokes [26]. In a Taiwanese 13-year cohort study on 486,890 patients with incident ischaemic stroke, SLE (n = 622) was independently associated with increased in-hospital mortality (adjusted OR 2.18, 95% CI 1.57–3.02) and higher risk of recurrent ischaemic stroke (HR 1.39, 95% CI 1.19–1.62) [26].

Although some studies report lower in-hospital mortality in SLE, it is largely due to younger age and milder stroke severity at presentation [27, 28]. SLE independently increases the risk of poor short-term prognosis in acute ischemic stroke (OR 5.94) [29].

Emerging evidence suggests that intravenous thrombolysis and mechanical thrombectomy can be safely and effectively performed in SLE patients with acute ischemic stroke [30, 31]. However, the heterogeneity of stroke mechanisms—including immune-mediated vasculitis, thrombosis, accelerated atherosclerosis, aneurysm formation, and small vessel disease—necessitates individualized and multidisciplinary management approaches [32, 33].

### Rheumatoid arthritis

RA is an independent risk factor of stroke [34]. A systematic review pooling data on 1,715,001 subjects demonstrated a significantly increased stroke incidence in RA patients (OR = 1.35; 95% CI 1.26–1.45) [34]. The risk was particularly high in women (OR = 1.6; 95% CI 1.19–2.16) and individuals above 65 years (OR = 1.24; 95% CI 1.02–1.5) [34].

A population-based study reported increased risks of both ischemic stroke (aHR = 1.47; 95% CI 1.36–1.58) and hemorrhagic stroke (aHR = 1.31; 95% CI 1.15–1.5) in RA [35]. Seropositivity in RA conferred higher cerebrovascular risk [35].

The excess stroke risk in RA depends on a complex interplay of systemic inflammation and vascular dysfunction [36]. RA-associated vasculitis affecting small- and medium-size cerebral vessels further predisposes to ischemic injury [36]. A genome-wide analysis revealed a significant positive genetic correlation between RA and stroke (rg = 0.3756), with five susceptibility genes (IRF5, RNASET2, ZNF438, UBE2LS, SYNGR1) converging on immune-inflammatory pathways [37]. Mendelian randomization data also demonstrated that genetically predicted RA increases adverse ischemic stroke outcomes (OR 1.09; 95% CI 1.01–1.18), whereas IL-6 inhibition reduces the risk (OR 0.88; 95% CI 0.77–0.99) [38].

RA patients present with enhanced subclinical atherosclerosis [39]. Carotid plaque prevalence is significantly higher in RA compared to non-RA controls [39]. Cardiac comorbidities further amplify stroke risk in RA. Nationwide Norwegian registry data showed that patients with atrial fibrillation and RA had a higher 5-year cumulative stroke incidence than those without RA (7.3% vs. 5%; HR 1.25; 95% CI 1.05–1.5) [40].

Established and RA-related risk factors may modulate stroke incidence. In the German RABBIT registry (12,354 RA patients and 166 reported strokes) age, hyperlipoproteinaemia, smoking, untreated cardiovascular disease, and infections were major determinants of stroke risk, while better physical function was protective against stroke [41].

Drug therapies exert variable effects on stroke risk in RA. TNFalpha inhibitor exposure was not associated with altered stroke incidence or post-stroke mortality [42]. A systematic review demonstrated that conventional synthetic DMARDs modestly increase stroke risk (OR 1.17; 95% CI 1.01–1.36), whereas tocilizumab reduces major adverse cardiovascular events without significantly affecting stroke incidence [43]. Biologic DMARDs did not significantly modify stroke risk, while targeted synthetic DMARDs showed a nonsignificant trend toward reduction [35]. In contrast, JAK inhibitors were associated with increased stroke (adj.ROR 1.25; 95% CI 1.16–1.34), particularly with upadacitinib and baricitinib [44]. Adherence to antimalarial therapies reduces stroke risk by 55% (aHR 0.45; 95% CI 0.36–0.58) [45].

Overall, suppressing inflammation, minimizing glucocorticoid exposure, and optimizing cardiovascular risk factor management are essential for cerebrovascular prevention in RA [46].

### Systemic sclerosis

SSc is an autoimmune disease characterized by immune dysregulation, progressive fibrosis, and widespread vasculopathy. Although traditionally considered a microvascular disorder, accumulating evidence indicates that SSc also involves macrovascular structures, predisposing patients to significant cardiovascular and cerebrovascular complications [47]. Neuroimaging and angiographic studies have demonstrated cerebral hypoperfusion and white matter hyperintensities in patients with SSc [47].

A systematic review of 17 studies including 6,642,297 subjects demonstrated that patients with SSc have a 64% higher risk of stroke compared with non-SSc controls (HR 1.64; 95% CI, 1.35–2.01) [48]. In a cohort study of 4,545 U.S. veterans with SSc (83% males; mean age 60.9 years), the incidence of ischemic stroke was higher among patients with SSc (15.3 vs. 12.2 per 1,000 person-years) [49].

Analyses of 525,570 ischemic stroke admissions from the 2016–2017 National Inpatient Sample identified 410 patients (0.08%) with SSc [50]. Although SSc confers an increased long-term risk of ischemic stroke, it does not significantly influence short-term clinical outcomes or resource utilization during acute stroke hospitalization [50].

### Ankylosing spondyloarthritis

AS is increasingly recognized as a disease associated with elevated cardiovascular and cerebrovascular risk [51]. Stroke incidence in patients with AS exceeds the same of the general population [52]. Although the risk rises with advancing age, younger patients with AS may also be vulnerable to ischemic stroke [52]. A systematic review of 11 studies involving approximately 1.7 million subjects demonstrated a 56% increased risk of stroke among patients with AS (HR 1.56, 95% CI: 1.33–1.79), with an elevated risk for ischemic stroke (HR 1.46, 95% CI 1.23–1.68) [51]. Another systematic review of 12 AS cohorts found a statistically significant increase in stroke risk compared with the general population (RR 1.21; 95% CI 1–1.47) [53]. In a German cohort study of 29,106 patients (mean age 54.8 years; 65% females), AS was associated with a significantly higher risk of transient ischemic attack (HR = 1.62, 95% CI 1.08–2.44) compared with matched controls [54]. A Mendelian randomization analysis using genetic data from 1,462 AS cases (FinnGen) and 40,585 stroke cases (MEGASTROKE) found no significant association between genetically predicted AS and overall stroke risk (OR 1.014; 95% CI 0.999–1.031) [55].

### Systemic vasculitides

Cerebrovascular involvement represents a serious and yet incompletely characterized complication of systemic vasculitis [56]. In the DCVAS cohort of 4,828 patients with newly diagnosed vasculitis, cerebrovascular events were identified in 3.5% of cases (n = 169; 95% CI 3–4.06), comprising stroke (2.13%) and transient ischemic attack (1.68%) [56]. The burden was highest in Behçet disease (BD) (9.5%), polyarteritis nodosa (6.2%), and Takayasu arteritis (6%), and lowest in IgA vasculitis (0.4%) and ANCA-associated vasculitides (~ 2%) [56].

Among systemic vasculitides, BD confers the highest cerebrovascular risk. In a systematic review including 9,813 patients with BD and 41,802 controls, BD was associated with an increased risk of stroke (aHR 2.083; 95% CI 1.339–3.24) [57]. A Korean cohort of 5,576 BD patients and 27,880 matched controls demonstrated an increased risk of stroke (HR 1.65; 95% CI 1.09–2.5) [58]. In a Taiwanese cohort of 306 newly diagnosed BD patients and 1,224 matched controls, ischemic stroke occurred in 4.2% versus 1.6%, respectively, over 10 years [59].

Neuro-BD occurs in approximately 5% of BD patients and typically develops years after systemic manifestations [60]. Neuro-BD is categorized into parenchymal and non-parenchymal forms [61]. Parenchymal disease affects the brainstem, cerebral hemispheres, or spinal cord and may present as meningoencephalitis [61]. Non-parenchymal disease predominantly involves the vasculature, manifesting as dural sinus thrombosis, arterial occlusion, or aneurysm formation [61].

Neurological manifestations may precede classical BD features [62]. A case report described a 39-year-old man with recurrent ischemic and hemorrhagic strokes over 11 months before developing typical BD manifestations; brainstem imaging findings were consistent with parenchymal Neuro-BD, and HLA-B51 positivity supported the diagnosis [62]. Likewise, a 38-year-old man presented with acute focal deficits, and further evaluation revealed small-vessel vasculitis, intracranial arteritis, and elevated interleukin-8 levels [60]. Immunosuppressive therapy resulted in complete recovery without recurrence [60]. Another report of a 40-year-old African American woman with visual impairment, recurrent strokes, and genital ulcers further illustrates the diagnostic challenges of BD in low-prevalence populations [63].

Stroke risk is high in ANCA-associated vasculitis (AAV) [64]. A systematic review of six cohort studies demonstrated a twofold increased stroke risk in AAV compared with non-AAV populations (pooled RR 2.02; 95% CI 1.02–4) [64]. A study on 325 patients with AAV reported 25 strokes (8%) during 2,206 person-years of follow-up, corresponding to an incidence rate of 11.3 per 1,000 person-years (95% CI 6.9–15.8) [65]. Stroke risk was significantly elevated among patients younger than 65 years (SIR 3.19; 95% CI 1.53–5.88) [65].

In a Korean cohort of 1,905 newly diagnosed AAV patients, stroke accounted for 64.6% of vascular events [66]. AAV was associated with a twofold higher risk of stroke (HR 2), consistently observed across microscopic polyangiitis, granulomatosis with polyangiitis, and eosinophilic granulomatosis with polyangiitis [66].

In a nationwide cohort of 2,644 patients with incident systemic necrotizing vasculitis (SNV), 159 (6%) experienced stroke, with the majority occurring within the first year of diagnosis (67.3%) [67]. The overall stroke risk in patients with SNV was higher than in the general population (standardized incidence ratio [SIR] 8.42) [67]. Among SNV subtypes, microscopic polyangiitis (MPA) demonstrated a greater stroke incidence than polyarteritis nodosa (adjusted incidence rate ratio [IRR] 1.98) [67].

Among large-vessel vasculitides, Takayasu arteritis (TA) represents a chronic inflammatory disease predominantly affecting women [68]. Cerebrovascular involvement can be an initial manifestation in TA [68]. A case report described a 45-year-old woman with simultaneous ischemic stroke and myocarditis, illustrating the potential for atypical and severe cardiovascular complications in TA [68].

### Stroke prevention in autoimmune rheumatic diseases

Preventive strategies in SLE should prioritize aggressive control of modifiable cardiovascular risks, including arterial hypertension and diabetes mellitus, smoking cessation and lipid management [69]. High disease activity and persistent systemic inflammation have been associated with increased cerebrovascular risk in SLE patients [7]. Lipid-lowering therapy, particularly statin treatment, is an important component of cardiovascular risk reduction and has been recommended in recent guidelines for the primary prevention of stroke [70]. Hydroxychloroquine confers additional antithrombotic benefit and remains a cornerstone of therapy [7]. Management of acute and recurrent strokes in SLE should include antiatherosclerotic interventions and targeted immunosuppressive and anticoagulant therapies [7]. Monitoring and targeting inflammatory markers and antiphospholipid antibodies holds promise for reducing stroke incidence and improving post-stroke outcomes in lupus and other ARDs [71].

Available evidence points to the benefits of targeted antiinflammatory therapy [72]. In a Taiwanese cohort, biologic DMARD therapies were associated with substantially reduced stroke incidence when compared with conventional synthetic DMARDs (2% vs. 5%) [72].

Cerebral rheumatoid vasculitis may present with ischemic stroke [72]. Combined rituximab and corticosteroid therapies have been reported to prevent recurrent cerebral strokes in RA and aid in post-stroke recovery [73].

Glucocorticoids remain central to the management of vasculitides, although optimal dose tapering is still a big issue [74]. Vasculitis patients with delayed tapering often suffer from vision loss and stroke (43% vs. 14%, respectively) [74].

Integrated approaches combining rheumatic disease control, optimized immunosuppressive therapies, and structured rehabilitation are essential to improve both stroke prevention and post-stroke outcomes.

### Post-stroke rehabilitation in autoimmune rheumatic diseases

Post-stroke rehabilitation outcomes vary among patients with ARDs. In a cohort of 47,853 stroke patients, including 368 with RA and 119 with SLE, patients with RA had worse functional recovery [75]. Although patients with SLE were younger than non-RA/SLE subjects (mean age difference 17.5 years), they demonstrated similar functional outcomes [75]. However, nationwide data indicate that patients with SLE have a significantly higher risk of functional dependency three months after ischemic stroke, despite similar stroke severity and access to rehabilitation [76]. One-year mortality following ischemic stroke and early mortality after hemorrhagic stroke are also increased in SLE [76].

Osteoarthritis (OA), while not autoimmune disease, also negatively influences post-stroke recovery. Pain, joint stiffness, mobility limitations, and constraints on optimal OA therapy may reduce participation in rehabilitation programs, thereby limiting functional gains [76]. Stroke survivors with OA have been shown to experience longer inpatient stays and lower levels of functional independence [77]. Additionally, knee pain and OA severity are weakly but significantly associated with slower ambulatory improvement, underscoring the additive burden of degenerative joint disease in stroke rehabilitation [77].

## Conclusion

Stroke represents a significant and multifaceted complication across ARDs. The pathogenesis reflects a complex interplay between systemic inflammation, immune-mediated vascular injury, established cardiovascular risk factors, and disease- or therapy-related adverse effects. Ischemic and hemorrhagic strokes in autoimmune rheumatic diseases occur at younger age, often with worse functional outcomes.

Early identification and aggressive management of modifiable risk factors, including arterial hypertension, dyslipidemia, and hypercoagulable states, combined with targeted anti-inflammatory and immunomodulatory therapies, are essential for stroke prevention. Post-stroke functional recovery is influenced by underlying autoimmunity. More research studies are warranted for stroke risk stratification and validating innovative imaging technologies to support personalized prevention, treatment, and rehabilitation strategies.
